# A retrospective study of the accuracy of Invisalign Progress Assessment with clear aligners

**DOI:** 10.1038/s41598-023-36085-5

**Published:** 2023-06-02

**Authors:** Bo Li, Yi-Meng Xu, Rui-Ying Shi, Yi-Rong Hu, Si-Ying Liu, Ze-Xu Gu

**Affiliations:** State Key Laboratory of Military Stomatology, National Clinical Research Center for Oral Diseases, Department of Orthodontics, School of Stomatology, The Fourth Military Medical University, Shaanxi Clinical Research Center for Oral Diseases, Xi’an, 710032 China

**Keywords:** Diseases, Medical research

## Abstract

The objective of this study was to detective the accuracy of model superimposition and automatic analysis for upper and lower dentition width in Invisalign Progress Assessment during the process of clear aligners. 19 cases were included in this study. Pre-treatment dental cast (T0) and post-treatment dental cast after staged treatment (T1) were available for three-dimensional model superimposition. Subsequently, movements of maxillary teeth in the horizontal plane (cross-section) after staged treatment and width of upper and lower dentition were measured by three-dimensional model superimposition in the real world and Invisalign Progress Assessment separately. Consequently, the data collected from these two methods were compared. In Invisalign Progress Assessment, movements of maxillary teeth in the horizontal plane after staged treatment was 2.31 (1.59,3.22) [median (upper quartile, lower quartile)] millimeter (mm), while in three-dimensional model superimposition, the result was 1.79 (1.21,3.03) mm. The difference between the two groups is significant (*P* < 0.05). Intercanine width upper, intermolar width upper, intercanine width lower, and intermolar width lower were 36.55 ± 2.76 mm, 56.98 ± 2.62 mm, 28.16 ± 1.85 mm, 53.21 ± 2.72 mm separately in Invisalign Progress Assessment and were 36.48 ± 2.78 mm, 56.89 ± 2.58 mm, 28.05 ± 1.85 mm, 53.16 ± 2.64 mm separately in three-dimensional model analysis, which was no significant difference among these groups (*P* > 0.05). The data from Invisalign Progress Assessment was not in parallel with what was achieved from model superimposition with palate as a reference completely. The accuracy of model superimposition in Invisalign Progress Assessment needs further investigation, whereas the accuracy of model analysis in Invisalign Progress Assessment was accurate. Thereby, results from Invisalign Progress Assessment should be interpreted with caution by the orthodontist in the clinic.

## Introduction

Clear aligners has been gradually became the choice of doctors and orthodontic patients, since the introduction of Invisalign appliances (Align Technology) in 1998, due to its better aesthetics and comfort^1^. With the progress of materials and the application of attachments, the scope of treatment was no longer limited to mild malocclusion^2,3^. In recent years, there were much research on the advantages and disadvantages of clear aligners. Compared with fixed appliances, the prevalence and severity of apical root resorption were less in patients treated with clear aligners^4,5^, and clear aligners were better for periodontal health than fixed appliances^6^, whereas clear aligners showed poorer root control during extraction space closure^7^. In addition, previous studies showed that the mean accuracy of Invisalign for all tooth movements was 50%^8^, and the accuracy of intrusion of the maxillary and mandibular central incisors were 44.7% and 46.6%^9^, respectively. A prospective study conducted on 53 canines of 31 subjects assessed a mean accuracy for canine rotation of 35.8%^10^. Simon et al. revealed a high predictability (88%) of the distalization movement of upper molars^11^.

Compared with fixed appliances, clear aligners can be removed by the patient. So, better patient compliance is required for successful treatment, if not or the tooth movement speed does not match the movement design of the treatment plan, the teeth will not fit the clear aligners. If it cannot be detected in time clinically, severe cases of tooth extraction may have "roller coaster" effect^12^ and mesial inclination of molars. The interval of patients' subsequent visit should not be too long, and orthodontists should regularly evaluate the patient's tooth movement during the treatment process.

The iTero Element, manufactured by Invisalign, has launched the Invisalign Progress Assessment, designed to help orthodontists monitor patients' clinical outcomes. At the follow-up visit, iTero was used for one-stage digital oral scan, and the digital model obtained by oral scan was superimposed on the pre-treatment digital model of dentition in Invisalign Progress Assessment, to observe the movement of the patient's tooth after a period of treatment. The digital model obtained by oral scan can also be superimposed on the predicted model corresponding to the design of 3D treatment plan at this stage. In this way, orthodontists can evaluate whether the patient's tooth movement conforms to the design of the treatment plan. This function is more and more trusted by orthodontics.

While the results of the Invisalign Progress Assessment may not reflect the actual movement of teeth accurately. When we superimposed the post-treatment digital model of dentition after staged treatment onto the pre-treatment digital model of dentition, with palatal rugae as the reference marker of superimposition^13^, different degrees of movement of the maxillary molars were found. However, this movement did not present in Invisalign Progress Assessment. This may be due to the Invisalign Progress Assessment using undesigned moving teeth as a model overlap reference marker. To evaluate the accuracy of the Invisalign Progress Assessment, adult patients with clear aligners were selected as the research objects, the actual movement data of maxillary teeth on the horizontal plane after stage treatment were measured by superimposing actual 3D models of dentition, then the data were compared with those obtained by Invisalign Progress Assessment. To provide a reference for the clinical reasonable application of orthodontic doctors.

## Material and methods

A total of 19 adult patients (1 man, 18 women; mean age: 28.7 ± 5.35 years) were selected from those who started orthodontic treatment at the Department of Orthodontics, School of Stomatology, The Fourth Military Medical University using Invisalign clear aligners from January 2018 to January 2021. There were 4 cases of extraction of premolars. Inclusion criteria were (1) permanent teeth, second molars erupt, (2) good compliance, every 2 weeks to replace the one-step aligner, wear not less than 22 h a day, (3) good hard palate development and clear palatal rugae, (4) the crown was intact, without abnormal attrition and bad prosthesis. Exclusion criteria were (1) the morphological changes of palatine rugae caused by trauma and other reasons, (2) changes in crown shape due to restorative treatment, and (3) patients with periodontal disease or who had undergone periodontal surgery. Qualified pre-treatment dental casts and post-treatment dental casts after staged treatment were made by professionals using alginate film for all cases. A total of 124 teeth were measured. This project was approved by the ethical committee of The Fourth Military Medical University Hospital of Stomatology (IRB-REV-2022047). Informed consent was obtained from all participants and this project was carried out under the Declaration of Helsinki.

**Figure 1:**
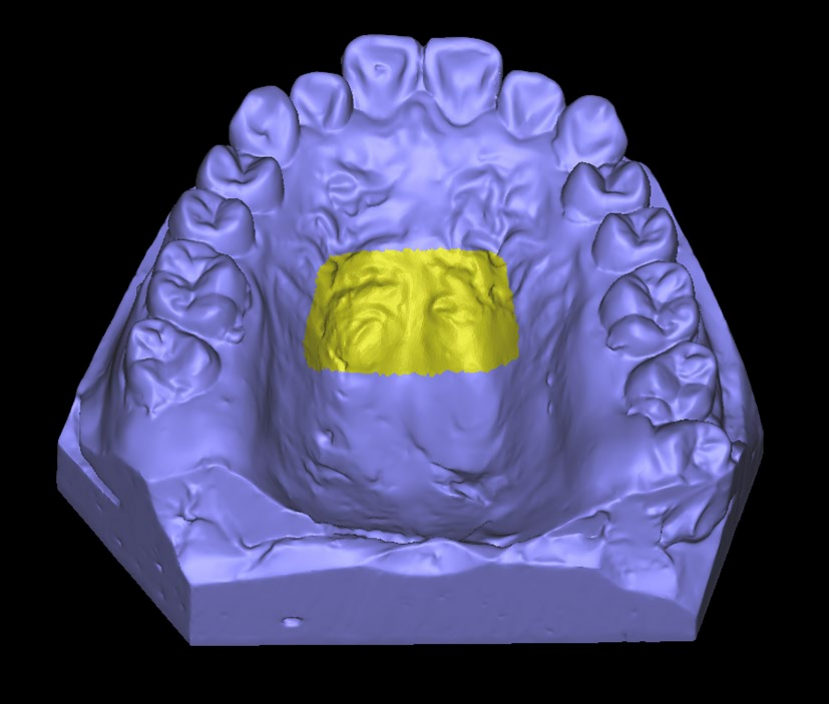
Schematic diagram of overlapping reference markers (third palatal fold and its distal portion).

**Figure 2:**
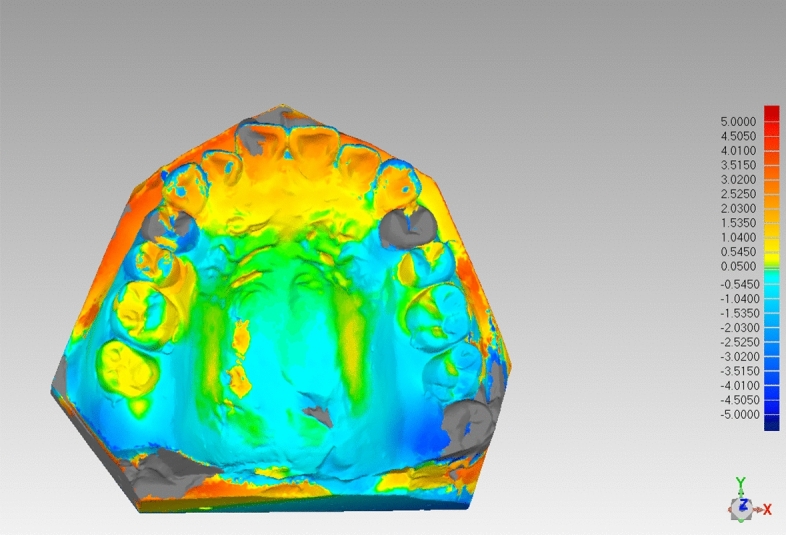
Deviation analysis (green indicates a high degree of overlap of palatal rugae area between two models).

The Model scanner R700 (3Shape, Denmark, No. Y14A004969) was used to scan the pre-treatment dental cast and post-treatment dental cast after staged treatment, and the digital model was obtained in stereolithographic files^14^. The stage was divided by the main tooth movement forms in this treatment stage, such as arch expansion stage, and anterior tooth retrusion stage. The digital models were imported into the MaterialiseProPlanCMF3.0 software. The pre-treatment digital model was set as a fixed object, and the stage digital model was set as a moving object, so as to carry out three-dimensional superimposition. The palatal rugae surface was selected as the mark surface for model superimposition (Fig. 1), and the superimposing model was exported. The superimposing model was then imported into Geomagic Studio 2013 (3DSystem, USA) software, and the model superimposition matching was detected using chromatographic deviation analysis (Fig. 2).

**Figure 3:**
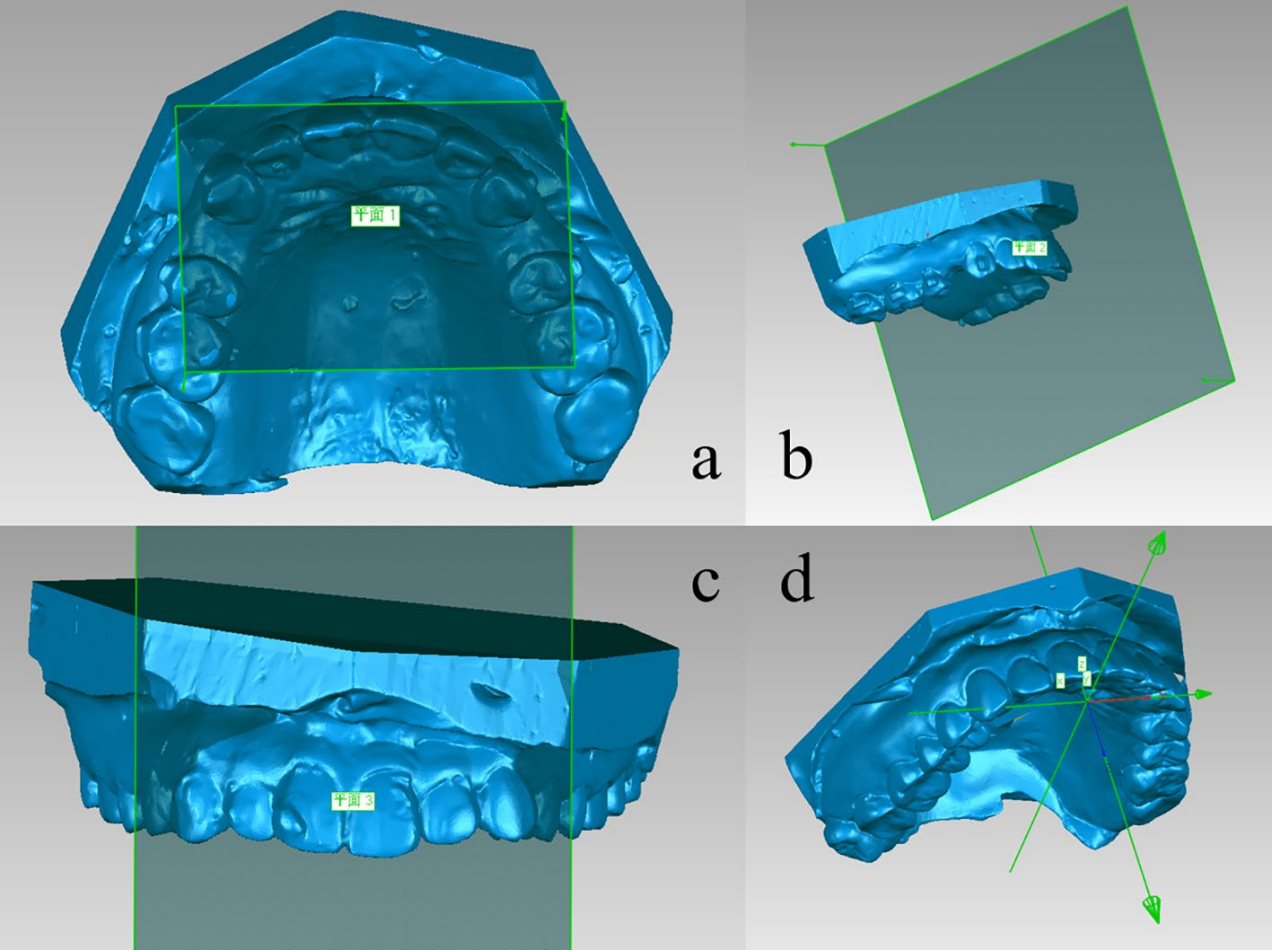
(**a**) XY plane (the point of the mesial incisor angle of the maxillary central incisor and the mesial palatal cusps of the bilateral maxillary first molars were used to fit the XY plane). (**b**) ZY plane (the ZY plane passes through the midpoint of the incisive papilla and bilateral palatal fovea and is perpendicular to the XY plane); (**c**) XZ plane (the XZ plane is perpendicular to the XY plane and ZY plane through the incisive papilla); (**d**) the three-dimensional coordinate system.

The global coordinate system was established using the Geomagic Studio 2013 (3DSystem, USA) software. The pre-treatment maxillary 3D digital model was taken as the reference model, and the origin of the coordinate system (projection of the incisive papilla on the XY plane), XY plane, ZY plane, and XZ plane were positioned. X-axis, Y-axis, and Z-axis represent transverse, sagittal, and vertical directions respectively, as shown in Fig. 3. In the anterior area, the midpoint of the incisal ridge and the cusp of the canine was selected as measuring marks. In the posterior area, one cusp that could be identified in the Invisalign Progress Assessment was selected for each posterior tooth as a measuring mark. The three-dimensional coordinates (X, Y,Z) of the measuring mark before and after stage treatment were obtained, denoted as pre-stage treatment (T0) and post-stage treatment (T1), and their coordinate differences (ΔX = X_T1_ − X_T0_, ΔY = Y_T1_ − Y_T0_, ΔZ = Z_T1_ − Z_T0_) represented the three-dimensional displacement of the measuring mark after stage treatment^15^. Calculate the projection movement data of the measuring mark on the XY plane (ΔL_XY_ = √(ΔX^2^ + ΔY^2^)), which represented the actual tooth movement data in the horizontal plane. The distance between the cusp of the two sides of the maxillary and mandibular canine was the width of intercanine, and the distance between the most protruding points of the buccal surface of the first molars was the width of intermolar.

**Figure 4:**
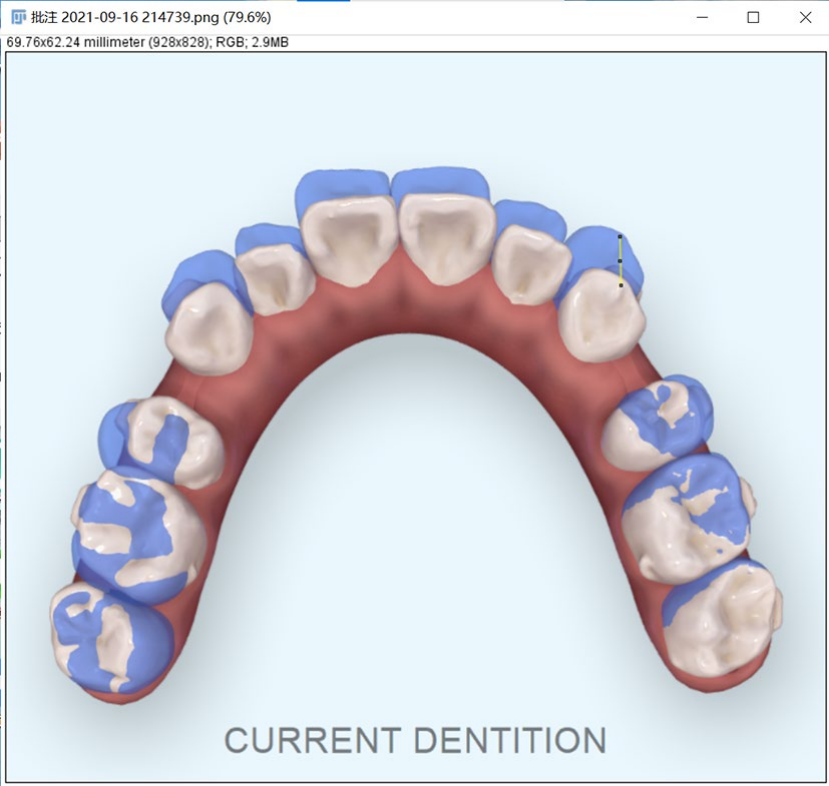
ImageJ measures the horizontal movement of the maxillary teeth.

After a period of treatment, stage dental casts were taken during the follow-up visit of patients, and the oral scan was performed to obtain the digital model of the current dentition, then the oral scan data were uploaded into Invisalign Progress Assessment. The model analysis data in Invisalign Progress Assessment were recorded, and the model was adjusted to the same Angle of the XY plane of the actual model superimposition, then screenshots were taken to obtain the horizontal plane image of maxillary dentition. Image analysis software ImageJ (National Institutes of Health, USA) was used to measure the horizontal distance of the measuring mark of the maxillary teeth (Fig. 4).

The obtained data were compared with the actual tooth movement data in the horizontal plane. If the Invisalign Progress Assessment showed that the tooth movement direction was opposite to the actual measurement, the tooth movement data in the horizontal plane measured by ImageJ was recorded as negative.

The measurement indexes and grouping are as follows. Invisalign Progress Assessment shows the horizontal movement distance of the maxillary, denoted as iTero group; Actual tooth movement distance in the horizontal plane of maxillary denoted as AM (Actual Movement) group; Maxillary intercanine width in Invisalign Progress Assessment, denoted as A1 group; Maxillary intercanine width of actual measured values, denoted as A2 group; Maxillary intermolar width in Invisalign Progress Assessment, denoted as B1 group; Maxillary intermolar width of actual measured values, denoted as B2 group; Mandibular intercanine width in Invisalign Progress Assessment, denoted as C1 group; Mandibular intercanine width of actual measured values, denoted as C2 group; Mandibular intermolar width in Invisalign Progress Assessment, denoted as D1 group; Mandibular intermolar width of actual measured values, denoted as D2 group.

### Statistical analysis

SPSS22.0 statistical software was used for statistical analysis. All models were measured by the same person. Two weeks later, 20% of patients were randomly selected and re-measured, and the intraclass correlation coefficient (ICC) was used for consistency analysis. *K–S* normality test was performed on all data, and *P* < 0.05 indicated that this group of data did not obey normal distribution (Table 1). As shown in Table 1, the iTero group and AM group do not obey normal distribution, so *Wilcoxon* rank-sum test was used for iTero group and AM group, and a t-test of paired samples was used for other groups. Data conforming to normal distribution are expressed as mean ± standard deviation ($\overline{X }$± *s*), and data that do not conform to the normal distribution are expressed as median (upper quartile, lower quartile). Test level *α* = 0.05, *P* < 0.05 was considered statistically significant.

**Table 1:** *K–S* normality test for each group of data.

Group	iTero*	AM*	A1	A2	B1	B2	C1	C2	D1	D2
*P* value	0.000	0.000	0.200	0.128	0.200	0.200	0.200	0.200	0.082	0.052

*P < 0.05, that is, this group of data does not conform to normal distribution.

### Ethical approval

This project was approved by the ethical committee of The Fourth Military Medical University, Hospital of Stomatology (IRB-REV-2022047).

## Results

### Consistency analysis results

In this study, the intraclass correlation coefficient (ICC) was used for consistency analysis. The test results were shown in Table 2, ICC of each group was greater than 0.75, indicating good consistency and reliable data measurement results.

**Table 2:** Consistency of retest with the same surveyor.

Group	iTero	AM	Actual model analysis
ICC	0.999	0.966	0.975

ICC > 0.75 indicates good consistency.

### Comparison between the actual horizontal tooth movement distance and the movement distance shown by Invisalign Progress Assessment

**Figure 5:**
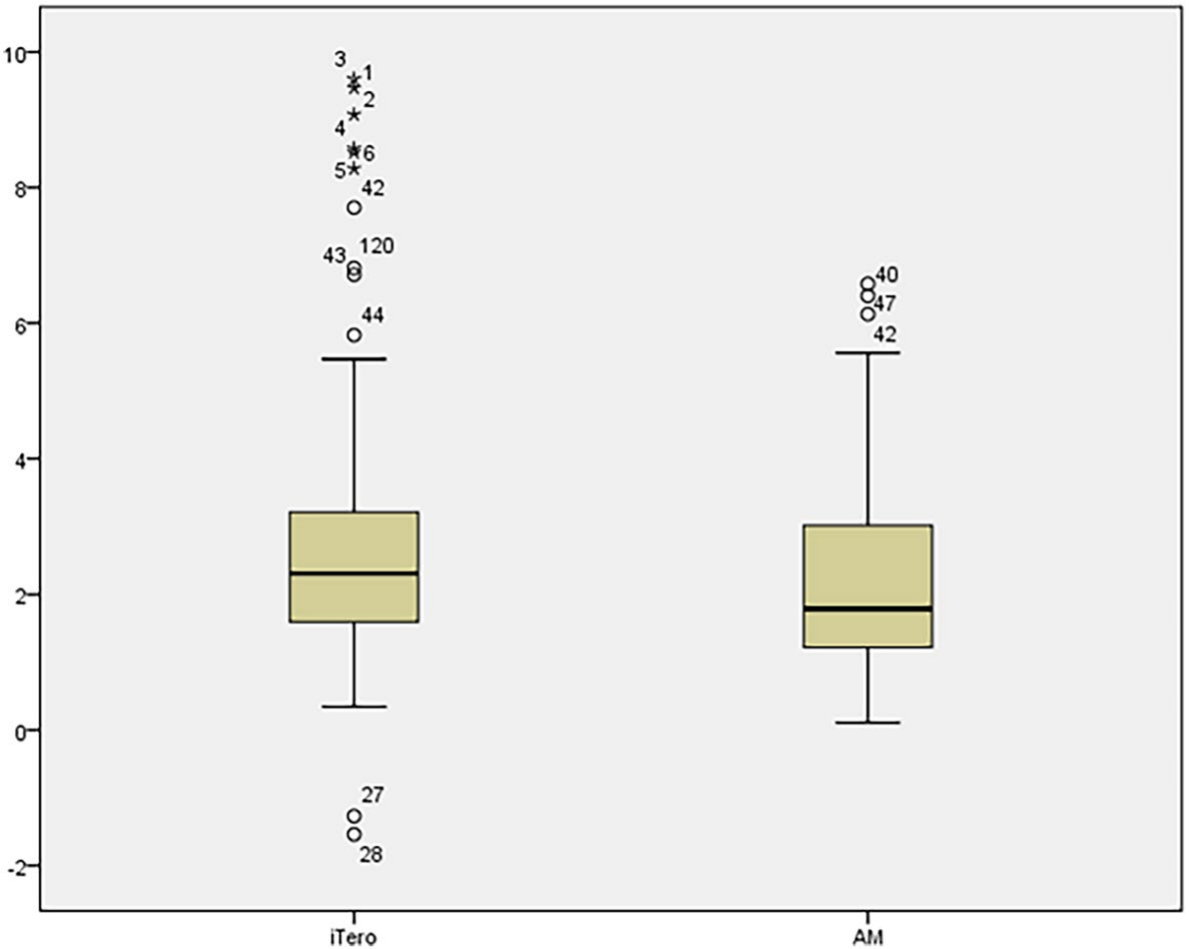
The box plot of horizontal movement distance of maxillary teeth in the iTero group and AM group.

Wilcoxon rank-sum test was used to compare the actual horizontal movement distance of maxillary teeth after a period of treatment with the movement of maxillary teeth distance shown by Invisalign Progress Assessment, *P* < 0.05, the difference was statistically significant (Table 3). The overall analysis showed that the actual distance of tooth movement was less than the movement value displayed by Invisalign Progress Assessment. The box plot was drawn for the two groups of data (Fig. 5), and the median, upper quartile, and lower quartile of the iTero group were all larger than those of the AM group.

**Table 3:** Comparison of horizontal tooth movement distance between T the iTero group and the AM group.

Group	iTero	AM	*P* value
Movement distance	2.31 (1.59,3.22)	1.79 (1.21,3.03)	0.000

Wilcoxon rank sum test was used, *P* < 0.05, and the difference between the two groups was statistically significant.

### Comparison of actual digital model analysis index data and Invisalign Progress Assessment model analysis index data

The index data analyzed by the initial and stage digital models and by the Invisalign Progress Assessment before and after stage treatment were compared, respectively. The results were shown in Table 4. There was no statistical significance in the index data of dentition width between the two groups (*P* < 0.05), and the correlation coefficients were all greater than 0.75.

**Table 4:** Comparison of model analysis index data (mm).

index	Sample size (n)	Invisalign progress assessment	Actual measurement	t value	*P* value
Maxillary intercanine width	35	36.55 ± 2.76	36.48 ± 2.78	1.853	0.073
Maxillary intermolar width	38	56.98 ± 2.62	56.89 ± 2.58	− 1.516	0.138
Mandibular intercanine width	38	28.16 ± 1.85	28.05 ± 1.85	− 1.883	0.068
Mandibular intermolar width	38	53.21 ± 2.72	53.16 ± 2.64	1.033	0.308

## Discussion

### Clinical application of Invisalign Progress Assessment

The Invisalign Progress Assessment can assist orthodontics in clinical diagnosis and treatment to more convenient, accurate, and efficient assessment of patients' oral dentition after staged treatment. An oral scan was performed at the time of the return visit, then the orthodontist can access the database and enter the functional interface of Invisalign Progress Assessment. The evaluation results were shown in different colors: (1) green indicates that the tooth movement was consistent with the 3D treatment design, (2) white indicates that the tooth has not been designed to move, and the tooth has not moved. (3) Yellow indicates that the tooth movement speed was slow and did not reach the expected position in the treatment design. (4) Purple indicates that the tooth movement direction was opposite to that of the treatment design. (5) Grey indicates that the tooth has not moved. And through the superimposition function, tooth movement can be compared and observed. Based on this, orthodontists can quickly evaluate the treatment effect of patients at this stage combined with clinical examination.

However, due to patent issues, Invisalign has not disclosed how it superimposes digital models in Invisalign Progress Assessment, and there were no literature to describe the accuracy of Invisalign Progress Assessment. Moreover, in the design of Invisalign 3D treatment design, the specific tooth movement value of each step in the three-dimensional direction cannot be read. Orthodontists can only conduct the qualitative evaluation by color, and specific results of tooth movement distance and movement mode cannot be obtained. So, the orthodontist cannot accurately evaluate the tooth movement effect.

### Actual tooth movement evaluation method for clear aligners

Currently, the digital model superimposition method was used to evaluate the actual movement effect of clear aligners. Studies have shown that there is no significant difference in accuracy between the digital model obtained by intraoral scanning and extraoral scanning technology and the dental model obtained by traditional impression technology^16–18^. Intraoral scanning technology was more efficient and comfortable, and the digital model was easy to store, so its clinical application was more and more extensive. Some scholars choose molars that have not been designed to move as overlapping markers for model superimposition to study the efficiency of tooth movement in clear aligners^19^. However, studies have found that the maxillary hard palate area is relatively stable during development^20^, and the model superimposition with the palatal rugae as an overlapping marker has better accuracy^21^. Vasilakos et al.^13^ had studied the accuracy and stability of different regions of the maxillary hard palate area as an overlapping marker, and found that the middle part of the third palatal rugae and its distal part have better three-dimensional superimposing accuracy and stability. In addition, some scholars have used Cone beam computed tomography (CBCT) combined with digital models to study tooth movement^22,23^. In this study, the maxillary third palatal rugae region was selected as reference marker, which has good accuracy and avoids the radiation exposure caused by CBCT.

### Accuracy of model analysis of Invisalign Progress Assessment

The results of this study showed that, compared with the actual digital model analysis, the results of model analysis displayed by Invisalign Progress Assessment had no statistical significance in the four groups of maxillary intercanine width, maxillary intermolar width, mandibular intercanine width, and mandibular intermolar width. The results indicates that Invisalign Progress Assessment has good accuracy in the analysis of dentition width. Model superimposition is not required for model analysis, and the measurement of width metrics is only related to the accuracy of intraoral and extraoral scans. The iTero scanner directly scans the patient's oral occlusion, and the scan result represents the patient's dentition information at this time. Therefore, clinical orthodontists can refer to the model analysis results to observe the treatment effect.

### Evaluation of digital model superimposition of Invisalign Progress Assessment

**Figure 6:**
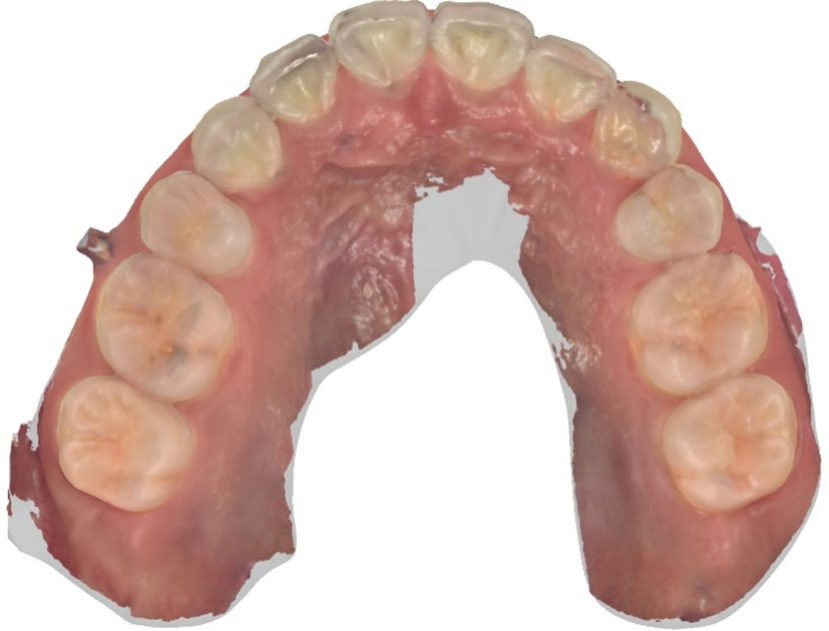
Intraoral scanning of the hard palate.

The results of this study showed that there was a statistically significant difference in the moving distance of teeth in the two-dimensional horizontal plane between the iTero group and the AM group (*P* < 0.05). Statistical analysis showed that the median, upper quartile, and lower quartile of the iTero group were all larger than those of the AM group (Table 3), that is, the actual tooth movements distance, such as the distance of molar distalization and anterior tooth retrusion, was smaller than the results displayed in Invisalign Progress Assessment. On the iTero platform, orthodontists can watch changes of teeth in each step of the treatment plan, but cannot export the 3D digital model of the stage design, only the initial digital model and the final digital model can be exported. Therefore, in this experiment, the horizontal image of the maxillary dentition was selected to measure the moving distance of the teeth in the horizontal plane. Since the vertical movement of the maxillary teeth in the experimental sample was not obvious, the vertical movement of the teeth was not included in this study. In addition, the maxillary hard palate data was not fully obtained when iTero was used for intraoral scanning (Fig. 6), and only the dentition model was used for Invisalign Progress Assessment, and the reference markers used for the digital model superimposition were unknown. Combined with the results of this study, it shows that the model fitting accuracy of Invisalign Progress Assessment needs to be further optimized. It is suggested that when the orthodontist uses the Invisalign Progress Assessment, even if the displayed result is green, it does not mean that the tooth movement conforms to the treatment design. In order to improve its accuracy, the hard palate can be scanned fully during the clinical oral scan, the algorithm can be improved, and the digital model can be superimposed with the hard palate as a marker, which can better represent the actual movement of the patient's teeth. Compared with traditional impression taking, direct intraoral scanning to obtain dentition data also improves patient comfort and clinical work efficiency, and reduces the loss of dentition information due to damage to the plaster cast^24^.

## Conclusion

1. Invisalign Progress Assessment has good accuracy in the analysis of dentition width.
2. Invisalign Progress Assessment was not completely consistent with the actual tooth movement of patients, and its accuracy was not consistent with model superimposition based on palatal rugae as a reference mark. Therefore, clinical orthodontists could not rely on Invisalign Progress Assessment completely, but should carry out analysis in conjunction with clinical examination.

## Data Availability

The datasets used and/or analyzed during the current study are available from the corresponding author on reasonable request.
